# Clinical outcomes and second-look arthroscopic findings of anterior cruciate ligament reconstruction with autograft, hybrid graft, and allograft

**DOI:** 10.1186/s13018-019-1439-8

**Published:** 2019-11-21

**Authors:** Xiaozuo Zheng, Yang Hu, Peng Xie, Tong Li, Yu-e Feng, Juyuan Gu, Shijun Gao

**Affiliations:** 1 Department of Orthopedics, Third Hospital of Hebei Medical University, No. 139 Ziqiang Road, Shijiazhuang, 050051 China; 2 Key Laboratory of Biomechanics of Hebei Province, No. 139 Ziqiang Road, Shijiazhuang, 050051 China; 3 The Second Department of Intensive Care Unit, Children’s Hospital of Hebei Province, No. 133 Jianhua Road, Shijiazhuang, 050030 China; 4 Department of Nuclear medicine, Third Hospital of Hebei Medical University, No. 139 Ziqiang Road, Shijiazhuang, 050051 China

**Keywords:** Anterior cruciate ligament, Reconstruction, Autograft, Hybrid, Allograft, Second-look

## Abstract

**Background:**

There is no consensus as to the choice of grafts for primary anterior cruciate ligament (ACL) reconstruction. The purpose of this study was to compare the clinical and second-look arthroscopic outcomes after ACL reconstruction by use of autograft, hybrid graft, and γ-irradiated allograft.

**Methods:**

Ninety-seven patients who underwent second-look arthroscopy after ACL reconstruction with autografts (28 patients, hamstring autograft), hybrid grafts (32 patients, hamstring autograft augmented with γ-irradiated tibialis anterior tendon allograft), or γ-irradiated allografts (37 patients, tibialis anterior tendons) were included in this study. The clinical outcomes were compared by using Lysholm score, International Knee Documentation Committee (IKDC) score, and Tegner activity score, and the side-to-side differences of KT-1000 measurement. Second-look arthroscopic findings were compared in terms of synovial coverage and graft tension.

**Results:**

There were no statistical significances among the three groups in Lysholm score, IKDC score, or Tegner activity score (*P* > 0.05). The KT-1000 examination showed more anterior laxity in the γ-irradiated allograft group than in the autograft or hybrid graft groups (*P* = 0.006, and *P* = 0.013, respectively). Two patients in the autograft group, 2 patients in the hybrid graft group and 4 patients in the allograft group were evaluated as graft failure on second-look arthroscopy. The synovial coverage was superior in the autograft group than that in the hybrid graft group or the allograft group (*P* = 0.013 and *P* = 0.010, respectively), and was comparable between the hybrid graft group and allograft group (*P* = 0.876). With regard to graft tension, the autograft group and hybrid group were comparable (*P* = 0.883) but showed better results than the allograft group (*P* = 0.011 and *P* = 0.007, respectively).

**Conclusion:**

The hamstring autografts and hybrid grafts used for ACL reconstruction produced equal efficacy but provided better knee stability than allografts. In addition, the hamstring autografts showed better synovial coverage than the other two graft types.

## Background

Anterior cruciate ligament (ACL) reconstruction is currently regarded as the best treatment for physically active patients with ACL rupture. A variety of autograft, hybrid graft, and allograft tissues are used for primary ACL reconstruction. Hamstring autograft is a popular choice due to the advantages of low donor site morbidity, early graft incorporation, and no risks of immune reactions and disease transmission [1, 2]. However, some patients may have small tendon diameters, which compromises the tensile strength of the grafts [3]. Clinically, this has translated to a higher likelihood of poor clinical outcomes as the graft diameter decreases. Previous studies have reported that the use of hamstring autografts with 8 mm in diameter or less resulted in increased graft failure risk and anterior knee laxity [4–6]. Allografts have been shown to be a reasonable alternative to small hamstring autografts. The major advantage of using allograft for ACL reconstruction is the availability of desired graft size with no donor site morbidities [7]. Some controlled clinical studies have reported comparable results with soft-tissue allografts and hamstring tendon autografts [8, 9], whereas others reported that the allograft tendons might have inferior graft maturity, higher risk of graft failure, and increased knee laxity than autograft tendons in ACL reconstruction [2, 10, 11]. Another solution to an inadequate graft diameter is hybrid graft which comprises autograft hamstrings and allograft soft tissues. It enables surgeons to customize graft size without harvesting additional autograft. However, only a few studies compared the clinical outcomes between hamstring autografts and hybrid grafts used for ACL reconstruction, and whether allograft augmented hamstrings are effective is still debatable. Leo et al. [12] and Li et al. [10] reported that the use of a hybrid graft has a comparable retear rate, knee stability, and patient-reported scores compared with the use of autograft hamstring. Jacobs et al. reported that allograft augmentation of hamstring autograft reduced the revision risk for young patients undergoing ACL reconstruction [13]. By contrast, other studies showed opposite results where inferior outcomes were observed for hybrid grafts compared with those of autografts [14–16]. Therefore, it is still unclear which of the three types of grafts, including hamstring autograft, hybrid graft, and soft-tissue allograft, is the optimal option for primary ACL reconstruction.

All biological tissues, whether allograft or autograft, undergo a similar ligamentization process when implanted as an ACL substitute [17]. Previous studies have reported that the quality of graft ligamentization has strong correlation with long-term survival of the grafts [18, 19]. Second-look arthroscopy is a less invasive method for evaluating the graft integrity and remodeling process of the reconstructed ACL [20–22]. Although some studies have reported second-look arthroscopic findings after ACL reconstruction, there is a paucity of research on the comparison of clinical outcome differences and graft morphology among different graft types.

The purpose of this study was to compare the patient-reported outcomes, knee stability, and second-look arthroscopic findings of patients who underwent ACL reconstruction with hamstring autograft, hybrid graft, or soft-tissue allograft. We hypothesized that the outcomes of ACL reconstruction with hamstring tendon autograft would have better results compared with those with hybrid grafts or allografts, and similar outcomes would be seen in the patients with hybrid grafts and allografts.

## Methods

### Participants

This study was carried out with the approval of the ethics committee of our institution. Patients who underwent second-look arthroscopy after anatomic single-bundle ACL reconstruction were retrospectively reviewed, and signed informed consent was obtained from each participant. The inclusion criteria were (1) primary ACL tears with single-bundle reconstruction; (2) the grafts with diameters equal to or more than 8 mm including hamstring autografts (combined gracilis and semitendinosus tendons), hybrid grafts (combined hamstring autograft and γ-irradiated tibialis anterior allograft), or γ-irradiated tibialis anterior allografts; (3) unilateral knee with no history of previous surgery; (4) skeletally mature patients aged more than 16 years old; (5) no signs of cartilage lesion and osteoarthritis; and (6) no combined ligament injury that requires surgical intervention. The exclusion criteria were (1) revision ACL reconstruction, (2) multiple knee ligaments requiring surgical intervention, (3) serious meniscal tear requiring total meniscectomy, and (4) patients who were unable to comply with the treatment protocol, or could not finish at least 12 months follow-up.

### Grafts preparation and surgical ACL reconstruction

For the autograft reconstruction, the semitendinosus and gracilis tendons were harvested and prepared as a 4-stranded or 6-stranded hamstring autograft. If the combined diameter of the autograft tendons was less than 8 mm, the γ-irradiated tibialis anterior allograft was used as augmentation to achieve a minimum desired diameter of 8 mm. In the allograft group, the γ-irradiated tibialis anterior allograft was prepared as a 2-stranded or 4-stranded graft. All the allografts were irradiated at a dose of 2.5 Mrad, and supplied by a certified tissue bank (Shanxi OsteoRad Biomateral Co., Ltd., Taiyuan, China).

Diagnostic arthroscopy was performed to identify the ACL tear. The combined meniscal injuries were addressed as needed before ACL reconstruction. The ACL remnant was generally preserved. All patients underwent anatomic single-bundle ACL reconstruction, with the femoral and tibial tunnel placed in the center of the femoral and tibial ACL insertion sites. The tunnel diameter was equal to the graft tendon diameter. The graft was then pulled into both tunnels from tibia to femur. The femoral side was fixed with a TightRope device (Arthrex, Naples, FL, USA), and the tibial side was fixed with an interference screw (Arthrex, Naples, FL, USA).

### Rehabilitation protocol

All patients followed the same rehabilitation protocol. The knee was immobilized with a long hinge brace immediately postoperatively for up to 8 weeks. Isometric quadriceps training was started immediately after surgery. Range-of-motion (ROM) exercise was started 2 days after the operation, with the goal of gaining normal ROM within 6 weeks. Partial weight bearing was allowed in the first 4 weeks, and full weight bearing was started at 8 weeks. Patients were allowed to run after 6 months, and return to sports activities gradually at 9 months after surgery. The patients were routinely followed up at 1, 3, 6, 9, 12 and 24 months, and clinical outcomes and second-look arthroscopic evaluation were recorded at the final follow-up postoperatively.

### Clinical evaluation

Subjective functional assessment included Lysholm score, subjective International Knee Documentation Committee (IKDC) score, and Tegner activity score. Knee stability was evaluated by the side-to-side differences of KT-1000 arthrometer measurement at 30-lb. force (MEDmetric, San Diego, CA, USA). The complications due to surgery were also recorded.

### Second-look arthroscopic evaluation

**Fig. 1:**
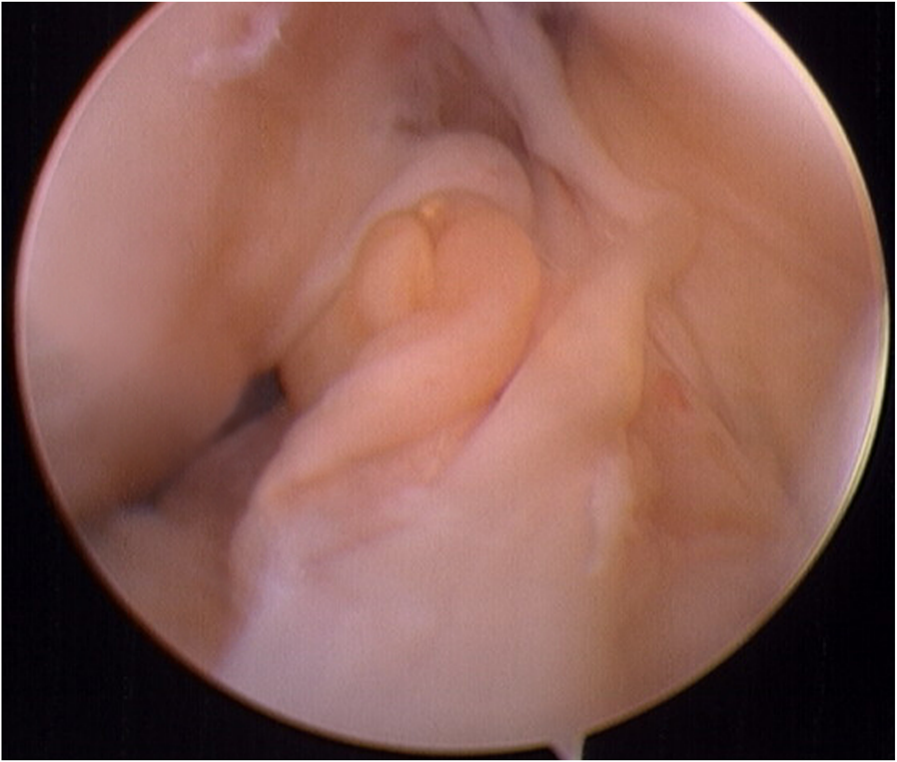
Biological failure defined as an atonic, disorganized, and non-viable ACL graft under second-look arthroscopy

**Fig. 2:**
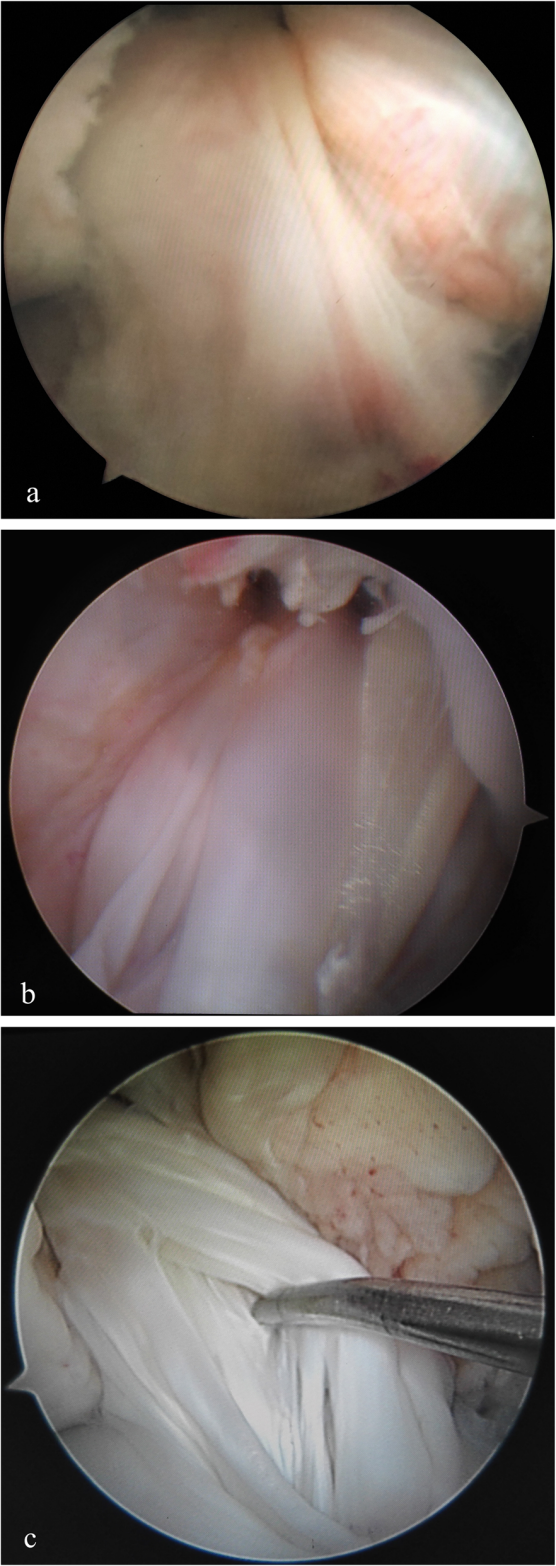
Arthroscopic classification of the reconstructed ACL graft based on synovial coverage. **a** Completely or more than 80% synovial coverage over the graft. **b** 50–80% of the synovial coverage over the graft. **c** Less than 50% or barely synovial coverage over the graft

Second-look arthroscopy was performed only in patients who requested femoral fixation device removal at least 12 months after ACL reconstruction. The purpose and risk of second-look arthroscopy were explained, and the informed consents were provided by all the patients. Graft failure was defined as a failure in the completion of the ligamentization process, leading to an atonic, disorganized, and non-viable graft [23] (Fig. 1). Graft healing in relation to synovial coverage, and graft thickness and tension were evaluated. Synovial coverage of the grafts was graded as “good” (completely or more than 80% covered), “fair” (50–80% covered), or “poor” (less than 50% or barely covered) [20, 24] (Fig. 2). Graft tension was evaluated using a probe with the knee in positions from extension to flexion. The middle part of the graft was manually measured and graded as “taut” (as tense as normal ACL), mildly lax (less tense than the normal ACL), and lax (complete tear or obvious loss of tension) [24, 25].

### Statistical analysis

The baseline characteristics of the three groups were compared using chi-square test for nominal variables, and analysis of variance (ANOVA) and least significant difference (LSD) test were conducted for continuous variables. The Kruskal-Wallis test and Mann-Whitney *U* test were used to test differences of the synovial coverage and graft tension among the three groups. Comparison of the knee functional scores and differences in KT-1000 were analyzed by ANOVA and LSD test. The level of significance was set at *P* < 0.05. SPSS software (version 19.0; SPSS Inc., Chicago, Illinois) was used for data analysis.

## Results

### Patient demographics

From January 2013 to February 2018, 97 patients who met the inclusion criteria and underwent second-look arthroscopy were enrolled, including 28 with autograft ACL reconstruction, 32 with hybrid graft ACL reconstruction, and 37 with allograft ACL reconstruction. There was no statistically significant difference in demographics at the time of initial ACL reconstruction among the groups (Table 1). The mean follow-up period of all the patients was 20.1 ± 6.7 months with no significant difference among the groups. The graft diameter was significantly larger in the hybrid group than the autograft and allograft groups (*P* = 0.000 and 0.012, respectively), but there was no significant difference between the autograft and allograft groups (*P* = 0.086).

**Table 1:** Demographic data of the study groups

Variable	Autograft	Hybrid Graft	Allograft	*P* value
Number of patients	28	32	37	–
Gender (male/female), *n*	23/5	20/12	26/11	0.243
Age, years	30.1 + 9.6	29.0 + 8.4	32.8 + 9.9	0.22
Side (left/right), *n*	7/11	13/19	20/17	0.426
Time from injury to surgery, weeks	9.4 + 7.1	10.8 + 8.1	10.4 + 6.9	0.746
Meniscal injury	16	16	23	0.595
Medial meniscus injury, *n*	8	5	8	
Lateral meniscus injury, *n*	5	6	11	
Medial and Lateral meniscus injury, *n*	3	5	4	
MCL injury, *n*	5	3	5	0.652
Graft diameter, mm	8.5 + 0.5	9.0 + 0.6	8.7 + 0.5	0.000
Follow-up, months	21.1 + 7.3	19.4 + 6.2	19.9 + 6.6	0.606

### Complications

There was no case of deep venous thrombosis, neurovascular injury, deep infection, and fixation failure in any group. One patient with wound disunion at the tibial incision area underwent debridement in the hybrid graft group. One patient with serious ROM deficit due to arthrofibrosis underwent surgical arthrolysis in the autograft group.

### Knee function and stability

No significant difference was found with respect to Lysholm score, subjective IKDC score, or Tegner activity score among the 3 groups at final follow-up assessments (*P* > 0.05) (Table 2).

**Table 2:** Knee functional assessment at final follow-up at final follow-up

Variable	Autograft	Hybrid graft	Allograft	*P* value
Lysholm score	87.7 + 8.5	86.8 + 10.2	85.5 + 8.0	0.588
IKDC score	81.9 + 9.3	82.1 + 8.9	79.9 + 8.6	0.544
Tegner activity score	6.0 + 1.7	5.9 + 1.6	5.5 + 1.6	0.385

The KT-1000 side-to-side differences at final follow-up were significantly inferior in allograft group than those in the autograft group and hybrid group (*P* = 0.006 and 0.013, respectively). Additionally, no significant difference was found between the autograft group and hybrid group (*P* = 0.748) (Table 3).

**Table 3:** Knee stability at final follow-up

Variable	Autograft	Hybrid graft	Allograft	*P* value
KT-1000	1.6 + 1.3	1.8 + 1.3	2.5 + 1.2	0.009

### Second-look arthroscopy

Two patients in the autograft group, 2 patients in the hybrid graft group, and 4 patients in allograft group were evaluated as graft failure on second-look arthroscopy. There were 1 patient with failed autograft, 1 patient with failed hybrid grafts, and 3 patients with failed allografts, and they underwent revision ACL reconstruction. The other patients did not received revision surgery because there was no feeling of instability during daily activities. With respect to synovial coverage over the graft, the autograft group showed significantly better results than the other two groups (*P* = 0.016 among the 3 groups, *P* = 0.013 for autograft group vs. hybrid graft group, *P* = 0.010 for autograft group vs. allograft group); however, there was no significant difference between hybrid graft group and allograft group (*P* = 0.876). With respect to graft tension, both the autograft and hybrid graft groups showed statistically significant differences compared with the allograft group (*P* = 0.008 among the 3 groups, *P* = 0.011 for autograft group vs. allograft group, *P* = 0.007 for hybrid graft group vs. allograft group), but no statistically significant difference was found between the autograft group and hybrid graft group (*P* = 0.883) (Table 4).

**Table 4:** Second-look arthroscopic evaluations of the study groups

Variable	Autograft (28)	Hybrid graft (32)	Allograft (37)	*P* value
Synovial coverage				0.016
Good	16	8	10	
Fair	9	16	16	
Poor	3	8	11	
Graft tension				0.008
Taut	14	17	7	
Mildly lax	10	10	19	
Lax	4	5	11	

## Discussion

The most important finding of our study was that the synovial coverage was significantly superior in the hamstring autograft group when compared with hybrid graft and γ-irradiated allograft groups. In addition, there was no difference between hybrid graft and allograft groups. Concerning the knee laxity evaluated by KT-1000 measurement and subjective graft tension under second-look arthroscopy, both the autograft and hybrid graft groups were significantly superior to those for patients in the allograft group. There were no significant differences in the Lysholm score, IKDC score, or Tegner activity score among the 3 groups during the short-term follow-up.

Hamstring autografts are commonly used for ACL reconstruction, with successful clinical results and low donor site morbidities [25, 26]. However, the recent literature suggests that a small graft diameter (especially those less than 8 mm) would biomechanically decrease the tensile strength [3], and clinically cause high revision risk and poor patient-reported outcomes [5, 6]. Unfortunately, the harvested hamstring tendons showed significant variability in size [27, 28], with 7 to 8 mm being most common for quadruple-stranded grafts [6]. Rather than harvesting additional autograft, surgeons attempt to augment the autograft with allograft tissue to create a hybrid graft. However, only a few studies investigated the clinical outcomes of hybrid ACL reconstructions, with no clear consensus on its effect. Burrus et al. [14] and Wang et al. [16] reported that hybrid hamstring ACL grafts led to poorer clinical scores and higher failure rates than autograft hamstring controls. On the contrary, other studies indicated that hybrid graft appeared to be a good treatment option, and hybrid grafts resulted in comparable or even better clinical outcomes and lower failure rates compared with hamstring autograft [12, 13, 29, 30]. Based on our findings, we did not find superior effects of ACL reconstruction with hybrid graft than that with autografts, although the graft sizes for the augmented group were significantly larger. The possible reason for this result could be that the irradiated grafts have lower tensile strength compared with nonirradiated or fresh grafts [3, 31, 32]. Therefore, the actually functional tensile strength of hybrid graft might be smaller than the homogeneous hamstrings, although the diameter was larger. This could also explain why the γ-irradiated allograft ACL reconstruction group could not achieve the same knee stability as homogeneous or augmented hamstrings group in this study.

It has been reported that the ACL grafts undergo a continuous remodeling process of ligamentization consisting of necrosis, revascularization, cellular repopulation, and collagen remodeling [33], and the quality of graft ligamentization is closely related to the grafts’ long-term viability [19]. Synovialization plays an important role in graft healing and is considered to affect survival of the graft [34]. Noh et al. [35] and Lee et al. [36] found that the extent of the synovialization is positively correlated with clinical results. The previous studies reported that hamstring autografts showed considerably better synovial coverage than soft tissue allograft based on second-look arthroscopic evaluation [22, 36, 37]. When compared with hybrid graft, hamstring autograft also showed better extent of synovial coverage after anatomic single-bundle ACL reconstruction [15]. The results of the current study are consistent with those previous studies in that superior synovialization was found with autografts than with allografts or hybrid grafts. Thus, it is reasonable to recommend a homogenous hamstring autograft with effective diameter for primary ACL reconstruction.

One interesting finding of our second-look arthroscopy was that the hybrid group did not show better synovialization than the allograft types, although autograft tissues acted as the main component in hybrid ACL graft. The possible explanation could be that the allograft portion in the hybrid implant was associated with increased inflammation, enhanced immunologic reaction, and slower histologic incorporation, which would slow down the synovialization and the graft remodeling process. Even at approximate 2 years after surgery, the allograft tendons might have inferior graft maturity than autograft tendons in ACL reconstruction [10, 22]. Therefore, we hypothesize that the advantages of increasing the implant size and mechanical properties through allograft augmentation would be compromised by the adverse effect caused by the allografts. Furthermore, the difference in knee laxity between the allograft group and hybrid graft group would diminish over time due to the similar ACL graft synovialization.

The failure rates of hybrid grafts and allografts were reported to be higher than that of autografts after primary ACL reconstruction [2, 14, 16, 38, 39]. However, we did not find significant difference in the graft failure among the three groups. There are several possible factors that led to this result. First, the graft failure was defined as “biological failure” based on arthroscopy in our study [23], which was different from symptomatic “clinical failure” [39] reported in most previous studies. Second, only the patients who underwent second-look arthroscopy were included in this study. The ACL retears based on physical examination, magnetic resonance imaging findings, and side-to-side arthrometer but without arthroscopic examination were not included for analysis. Third, some patient did not return to the full competitive activities at the finial follow-up due to the rehabilitation protocol. We hypothesize that the failure rate would increase in long-term follow-up, because the ACL grafts with poor maturation may not be able to survive intense sports.

## Limitation

There were several limitations in our study. First, the main areas that can be improved on in our future studies include the retrospective design, the limited sample size, and the short-term follow-up. Second, not all the patients who underwent the primary ACL reconstruction were included in our study. We conducted the analysis only on patients who underwent second-look arthroscopy after ACL reconstruction. Therefore, the selection bias could not be avoided. Third, it is quite difficult to evaluate the back of the ACL graft during arthroscopic surgery because it is located deeply in the knee joint. Forth, the assessment on synovial coverage and graft tension depended on the subjective judgment since no standard quantification method has been developed.

## Conclusions

There was no difference in patient-reported knee function among the outcomes of ACL reconstruction with hamstring autografts, hybrid grafts, and allografts in short-term follow-up. However, the hamstring autografts and hybrid grafts produced comparable knee stability and they both outperformed the allografts. The second-look arthroscopy revealed that hamstring autografts resulted in better synovial coverage than the other two graft types.

## Data Availability

The datasets used and/or analyzed during the current study are available from the corresponding author on reasonable request.

## Abbreviations

ACL: Anterior cruciate ligament
IKDC: International Knee Documentation Committee
ROM: Range-of-motion
